# Interdisciplinary, interprofessional, patient- and family-centered rounds: A systematic mapping review protocol of global interventions and outcomes

**DOI:** 10.1371/journal.pone.0357968

**Published:** 2026-10-06

**Authors:** Numair Belgaumi, Megan M. Coe, Benjamin Oelkers, Jordan Wrigley, Samantha Hayes, Michael Bashir, Anneliese Williams, Jocelyn Rivera, Dylan Graetz, Erin Abu-Rish Blakeney, Sara Malone, Asya Agulnik

**Affiliations:** 1 Department of Pediatrics, Stead Family Children's Hospital, University of Iowa Health Care, Iowa City, Iowa, United States of America; 2 Department of Global Pediatric Medicine, St. Jude Children's Research Hospital, Memphis, Tennessee, United States of America; 3 Biomedical Library, St. Jude Children's Research Hospital, Memphis, Tennessee, United States of America; 4 Department of Internal Medicine, Mississippi Baptist Medical Center, Jackson, Mississippi, United States of America; 5 Department of Pediatrics, Division of Emergency Department, Hospital Infantil Teletón de Oncología, Querétaro, Mexico; 6 Department of Biobehavioral Nursing and Health Informatics, School of Nursing, University of Washington, Seattle, Washington, United States of America; 7 School of Public Health, Washington University, St. Louis, Missouri, United States of America; 8 Division of Critical Care, St. Jude Children's Research Hospital, Memphis, Tennessee, United States of America; Li Ka Shing Faculty of Medicine, The University of Hong Kong, HONG KONG

## Abstract

Effective communication among healthcare disciplines, professions, and patients is crucial for delivering coordinated inpatient care and achieving improved patient outcomes. The practice of daily rounds has a long history, evolving over time in structure and in the individuals involved. Rounds increasingly include diverse healthcare professionals and, in some cases, patients and families. However, inconsistent terminology—such as interprofessional, interdisciplinary, multiprofessional, and multidisciplinary—complicates research and implementation of team rounding practices. Existing reviews have explored interprofessional and patient- and family-centered rounds but less frequently addressed interdisciplinary rounds and global settings. We will conduct a systematic mapping review to describe the landscape of literature on interdisciplinary, interprofessional, and patient- and family-centered rounds in hospital settings worldwide (PROSPERO CRD420250655348). We aim to examine how daily care rounds are defined, who participates, what frameworks are utilized, and where these practices are studied. Additionally, we will identify reported strategies for implementing rounds and measures used to assess outcomes on patients, families, clinicians, healthcare teams, and institutions. Following PRISMA-P guidelines, we will include peer-reviewed quantitative, qualitative, and mixed-methods studies published since 1990, with no geographic or language restrictions. Eligible studies must focus on daily inpatient rounds involving multiple professions, disciplines, and/or patients and families, and report outcomes or implementation strategies. Data will be extracted on study characteristics, rounding practices, interventions, outcomes, and barriers or facilitators to implementation. Study quality will be assessed using the Mixed Methods Appraisal Tool (MMAT). Our review will provide a comprehensive synthesis of global rounding practices, highlight gaps in definitions and implementation strategies, and inform future interventions to enhance team communication in diverse hospital settings. By capturing a broad range of studies, this work will support efforts to standardize and expand team rounds to improve hospital care coordination worldwide.

## Introduction

Caring for hospitalized patients requires coordinated input from individuals with varying roles and expertise. High-quality communication between healthcare disciplines and professions is needed to achieve this coordination [1]. Effective team communication improves patient outcomes and reduces adverse events, while ineffective communication is a known source of medical errors and patient harm [2]. Daily inpatient rounds are designed to convey information, establish plans, and set goals for hospitalized patients; and in many hospitals, they are the primary opportunity for daily team communication [3].

Daily rounding practices have evolved over time, at times focused on medical trainees, nurse-physician communication, and developing into a routine that includes multiple healthcare professions and an orientation towards patient- and family-centered care [4,5]. Incorporation of healthcare team members from different disciplines and professions into rounds has been shown to improve patient outcomes, healthcare team satisfaction, quality of care, and team collaboration [6]. Patient and family-member inclusion in rounds has also been shown to benefit healthcare outcomes and patient/family experience [7,8].

The terminology used to describe rounds that include additional health workers beyond medical doctors varies. Terms such as *interprofessional*, *interdisciplinary*, *multiprofessional*, and *multidisciplinary* are often used interchangeably, creating confusion and making research on rounds challenging to navigate [9]. In this review, we distinguish *professions* from *disciplines* using the following definitions. Professions have different training and certification – such as physicians, nurses, nurse practitioners, respiratory therapists, pharmacists, dieticians, and social workers. Disciplines are specialties within a profession, such as intensive care, oncology, cardiology, and neurology. While a detailed examination is beyond the scope of this study, a distinction has also been proposed between the “inter-” and “multi-” prefixes. *Interprofessional* and *interdisciplinary* rounds emphasize integration and active collaboration among team members [10]. In contrast, *multiprofessional* and *multidisciplinary* rounds involve the presence of these team members, but their contributions remain within traditional boundaries [10].

Previous scoping and systematic reviews of interprofessional and patient- and family-centered rounds explored their impact on outcomes such as quality of care, team collaboration, patient centeredness, implementation strategies, and patient/family involvement [6,7,11,12]. Three of these reviews were limited to articles in English, while one also included literature in Dutch and French. These reviews also had criteria limiting them to new implementations of specific rounds interventions or required formal research study designs. These strict criteria – and the overall lack of literature from low- and middle-income countries (LMIC) – resulted in only a handful of studies from LMICs in these reviews.

Previous reviews and studies do not make a distinction between inclusion of team members of different professions and disciplines. Several studies that use the term “interdisciplinary” describe interprofessional engagement in rounds [6,11]. Multiple medical specialists are often involved in a patient’s care team, especially in the case of complex conditions and critically ill patients [13]. Understanding the extent of interdisciplinary involvement in rounds is necessary to guide further study and intervention development.

Several studies have developed structured interprofessional and patient- and family-centered rounding frameworks, which provide insight into how rounds can be operationalized in practice and research [14–16]. Other literature has explored the implementation of tools and job aids to enhance the implementation and function of interprofessional and patient- and family-centered rounding [17,18], although these tools have not been systematically summarized or had their impact evaluated [14,16–19]. It is evident that, while a large body of research has been conducted on rounds, a lack of standardization persists in the use of common definitions, approaches to improvement and implementation, and outcome measures.

To address these knowledge gaps, we propose a systematic mapping review to understand the landscape of research on interdisciplinary, interprofessional, and patient- and family-centered rounds, and to examine how daily care rounds are defined, who they include, what frameworks are used, and where they are studied across global settings. Additionally, we aim to describe the evidence supporting existing strategies for implementing rounds and characterize the outcome measures used across multiple levels, including the patient/family, clinician, healthcare team, and institutional levels. Our review will fill several identified gaps in the literature. It will have no language restrictions and will include publications reporting research or quality improvement initiatives to optimize existing rounds, with the hopes of identifying literature with broader global representation. We will differentiate studies that report including multiple professions from those that report including multiple disciplines and will include studies reporting on patient/family centered rounds, regardless of other features of the intervention.

## Methods

### Protocol and registration

This protocol follows the Preferred Reporting Items for Systematic Review and Meta-Analysis Protocols (PRISMA-P) guidelines [20] and the Synthesis Without Meta-Analysis (SWiM) guidelines [21]. It was registered in the Prospective Register of Systematic Reviews (PROSPERO) on February 24, 2025 (CRD420250655348).

### Study timeline

Our initial search for evidence was conducted in September 2024, and screening was completed in October 2025. Data extraction is underway with expected completion by September 2026. When initial data extraction is nearing completion, we will conduct a gap analysis to identify publications since September 2024. Analysis and reporting of results is expected to be completed by late 2026.

### Protocol status and amendments

Prior to the search, the team drafted a protocol that outlined the key research questions, inclusion/exclusion criteria, and search strategy in collaboration with a medical librarian. The procedures were refined for clarity and completeness prior to PROSPERO registration and initiation of screening. The case report form was developed and refined to capture planned data elements. The decision to conduct a gap analysis was made due to the extended time elapsed since the search; while decisions to add PsycINFO and clarification of analyses were in response to peer review. These changes will be reflected in a PROSPERO amendment. No other changes were made to the research questions or overall review approach following PROSPERO registration. Any future modifications to the review protocol will be documented through a modification to the PROSPERO protocol and will be reported and justified in the manuscript.

### Eligibility criteria

We will include peer-reviewed quantitative, qualitative, and mixed-methods research, including ethnographies and quality improvement reports published after January 1, 1990. Abstracts, case reports/series, conference material, editorials, commentaries, opinion pieces, letters, protocols, and systematic or scoping reviews will be excluded. No geographic or language limitations on where the study was conducted will be used. Eligibility criteria are summarized in Table 1.

**Table 1:** Inclusion and Exclusion Criteria.

Category	Inclusion Criteria	Exclusion Criteria
**General Study Characteristics**	Peer-reviewed Study design: Quantitative, Qualitative, or Mixed Methods	Case report, Commentary, Protocol, Conference materials, Literature review
**Population**	Hospitalized Patients	
**Intervention**	Features of rounds: • Daily and on patient care • Interprofessional, Interdisciplinary, and/or patient- and family-centered (based on terminology or descriptions) • Rounds may be newly implemented, or an intervention to support rounds may be implemented (checklist, structural change, etc.)	Features of rounds: • Less frequent and/or focused on a specific topic (e.g., grand rounds, ethics rounds, infection prevention rounds)
**Outcome**	Any of the following: • Measurement of an outcome impacted by (or associated with) rounds (including process outcomes related to rounds) • Assessment of the barriers and facilitators of implementation • Presentation of a rounds-related intervention or tool developed through a reported process	No outcomes presented or assessment made.

### Population

The population of interest includes patients in any inpatient setting in which daily patient care rounds are conducted. Studies that focus on rounds that are not in the hospital setting will be excluded.

### Intervention

This systematic review aims to assess how research has been conducted on interdisciplinary, interprofessional, and/or patient- and family-centered inpatient rounds. Rounds must be described as either interdisciplinary, multidisciplinary, interprofessional, multiprofessional, patient- and family-centered, or a combination of the three. To be included, rounds must be conducted on hospitalized patients, discuss patient care, and be conducted daily. Other types of rounds, such as grand rounds, Schwartz rounds, weekly rounds, or ethics rounds, will be excluded. Rounds are defined as any practice that brings disciplines, professions, and/or families together with the purpose of discussing the care of hospitalized patients. Studies using the terms “multidisciplinary” or “interdisciplinary” as descriptors for rounds will be included, or if the study describes rounds including multiple medical subspecialty physicians. Similarly, studies will be included if terms such as “multiprofessional” or “interprofessional” are used as descriptors for rounds, or the study describes rounds participants who are healthcare professions other than physicians. This includes professions such as nurse practitioners, nurses, pharmacists, respiratory therapists, dietitians, case managers, and social workers. Patient- and family-centered is defined as the inclusion of the patients, family members, and/or caregivers during rounds.

Interventions to support the initiation or improvement of rounds include a wide variety of activities and implementation strategies. These may include changes to the format or structure of rounds, the introduction of checklists or other tools, or provision of educational resources for patients or healthcare professionals. Included studies may describe initial implementation of these rounds-related interventions, efforts to improve the practice of rounds, or ongoing assessment of rounds.

### Outcome

The included studies will describe an outcome related to rounds. This will be interpreted broadly to include studies that report the association or effect of rounds or a rounds-related intervention on patient, family, health provider, and/or service outcomes, either quantitatively or qualitatively. We will also include studies reporting barriers and facilitators of rounds implementation or other implementation outcomes. Finally, manuscripts that describe the development of a rounds-related intervention or support tool will be included.

### Data sources and search strategy

The search strategy was developed in collaboration with a systematic review librarian and tested against a pilot set of exemplar articles (n = 10) for comprehensiveness. The search will be translated and executed in EMBASE (via Elsevier), MEDLINE (via PubMed), CINAHL (via EBSCO), Web of Science Collections (including SciELO), Global Health (CABI), Sociological Abstracts, PsycINFO, and ERIC. The search contains a mix of controlled vocabulary or subject heading (e.g., Medical Subject Headings or MeSH) and keywords for “multiprofessional”, “interprofessional”, “multidisciplinary”, “interdisciplinary”, “rounds”, “hospital”, “bedside”, and “inpatient”. There were no language restrictions and the search identified articles published after January 1, 1990. The final search strategy is outlined in S1 File.

We will use Covidence, a web-based systematic review platform, to facilitate title/abstract screening, full-text review, and conflict resolution [22]. Covidence will identify duplicate entries for removal.

### Study selection and screening process

Titles and abstracts of studies identified in the search will be screened in Covidence. Study team members will undergo training on the use of Covidence and criteria for inclusion and exclusion, and then complete a knowledge check activity before initiating independent screening. The team of reviewers will include bilingual individuals who are fluent in languages including Spanish, German, and Chinese. Through a global network of collaborators, we will identify individuals fluent in additional languages, as needed. These individuals will either complete all training and review independently or read and discuss the source in detail with a trained study team member to make decisions.

Each title/abstract will be independently screened by two reviewers using the described eligibility criteria. Conflicts in screening will be resolved by a third reviewer. Periodic team meetings will be held to continually guide reviewers and discuss more complex examples to help improve screening quality.

Full-text articles will be obtained through a rigorous process that includes searching through at least two academic institutions, requesting through interlibrary loan, purchasing articles directly from publishing journals, and contacting corresponding authors. Titles and abstracts for which full text cannot be obtained through this process will be excluded. Full-text screening will use the same criteria as title/abstract screening and will be guided by a project-specific inclusion/exclusion flowchart (Figure 1). Reviewers will record an exclusion reason for excluded full-text articles (e.g., wrong publication date, wrong study type/design, wrong setting, incorrect rounds type, not multidisciplinary, multiprofessional, or patient- and family-centered, and no outcome or assessment).

**Fig 1:**
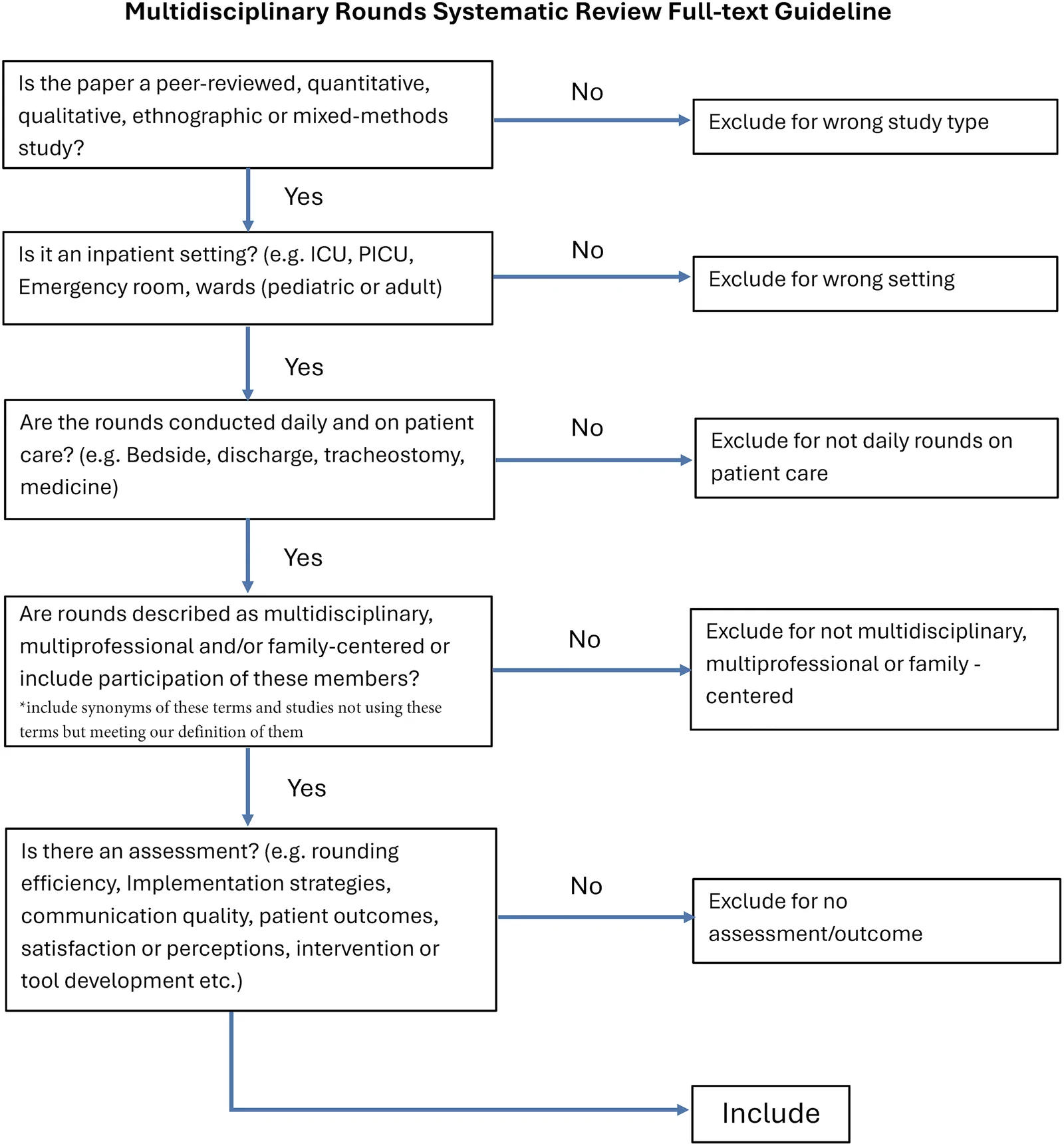
Full-text review flow chart.

Flow chart describing the process for full-text review and application of exclusion reasons.

### Data extraction and management

A data extraction tool will be developed in REDCap, a secure web-based platform for research data capture [23], and refined through testing by at least four research team members. Data extraction will include general study characteristics, description of rounds and any intervention, description of assessed outcomes, and appraisal of methodologic quality. Extracted elements are summarized in Table 2 and presented in full in S2 File.

**Table 2:** Summary of data extraction tool. The data extraction tool has four instruments. This table includes examples of data collected on each instrument.

Instrument	Examples of Data collected
General Study Characteristics	• publication year • country • hospital type • hospital unit • patient population • study objective • study methods
Rounds and Intervention	• terms used by study authors to describe rounds (interprofessional, multidisciplinary, family-centered, etc) • team composition of rounds (specific professions, disciplines, family included – based on descriptions of rounds) • study intervention (goals, changes made)
Outcomes	• categories of outcomes (patient, team, rounds-specific, implementation, etc.) • specific outcomes measured • for each outcome: • methods • direction of change • specific findings • implementation barriers and facilitators
Quality Assessment	• Appraisal based on type of study (using Mixed Methods Appraisal Tool [24])

Each study team member will be required to view a training, complete data extraction of two training articles, and review and respond to training article feedback before independent data extraction can begin. One study team member will independently extract data from each included article, and a second team member with review and validate all extracted data. The two reviewers will discuss discrepancies and consult a third team member to resolve conflicts, if needed.

In addition to general information about the study setting and methods, the study objectives will be categorized as one or more of the following:

1. Develop or implement a new team rounding process, change in team rounding process, rounding support tool, and/or strategies to support implementation
2. Assess the association between rounds or a rounding intervention on an outcome of interest
3. Assess determinants of team rounds implementation success or failure (barriers and facilitators)
4. The main objective of the study is not related to rounds (rounds are part of a multicomponent “parent” intervention, rounds is the setting to study something else)

Characteristics of rounds will be collected, including who is included, terminology used to describe rounds, location where rounds are conducted, and the use of any evidence-based rounds framework. In addition, we will capture the types of changes made to rounds through interventions, tools, and other changes implemented in the studies. When a change to rounds is part of a multicomponent intervention, the aim of that intervention will be collected in addition to details of the rounds-specific element.

We will capture the categories (patient, team, service, implementation, etc.) of measures presented as associated with or an outcome of rounds or a rounding intervention. Within each category, we will record the type of data collection used (quantitative and/or qualitative) and the findings of each specific outcome. For studies that include an assessment of barriers and facilitators to implementation of rounds or a rounds intervention, the specific determinants of implementation and frameworks used to identify these determinants will be extracted.

### Appraisal of study quality

Two study team members will assess the methodologic quality of each study using the Mixed Methods Appraisal Tool (MMAT) [24,25]. The MMAT tool is appropriate for a review such as this, which includes various types of studies and does not intend to assess causal effectiveness. Each article’s methodology will be identified as being qualitative, quantitative, or mixed methods. Quantitative studies are further classified as randomized controlled trials, non-randomized, or descriptive. The MMAT asks five methodological quality questions relevant to each study type, which are answered “Yes”, “No”, or “Can’t Tell”. When the response to an item is “No” or “Can’t Tell”, the appraiser will comment on their rationale. All data extractors will be trained on the use of the MMAT tool and provided a user guide for the MMAT.

### Data synthesis and analysis

General study characteristics (publication year, country of origin, language, publishing journal, and year), study designs, hospital setting (hospital type, ward, ICU, subspeciality inpatient unit), and patient population (pediatric, adult) will be analyzed descriptively and presented in tables. Similarly, characteristics of rounds including terminology used (interprofessional, multidisciplinary, etc.), location of rounds, and team composition of rounds (professions, disciplines, and/or family inclusion) will be analyzed and presented in tables.

Rounds-related interventions and implementation strategies developed and/or tested in the included studies will be described and categorized by the type of changes made to rounds and the frameworks used. We will quantify the measures used to assess the outcome of rounding interventions. Within categories of similar interventions, we will summarize the mixed-methods findings for the reported outcomes in tables and plots, including describing the magnitude and range of observed quantitative associations [26] and themes identified in qualitative findings. This approach will allow us to review and describe the reported outcomes of rounds interventions, including identifying interventions with limited data, which may be amenable to further study. If intervention categories include many studies, we will also describe subgroups by unit type and region. MMAT criterion ratings will be presented to summarize the quality of the included studies.

Quantitative descriptive analyses will be conducted in R [27] and Microsoft Excel. Given the variation in rounds-related interventions and study methodologies, we do not expect to conduct a formal meta-analysis of outcomes. Formal quantitative risk of bias assessment will not be conducted, however potential publication bias will be considered qualitatively during interpretation of findings. Confidence in cumulative evidence will be assessed qualitatively considering (1) methodological quality (MMAT), (2) consistency of study findings across methods, and (3) adequacy of the evidence base.

## Anticipated results

This systematic review will identify the breadth of literature reporting on the development and implementation of team rounds as well as the specific interventions and outcomes reported. This work will broaden our understanding of how team rounds are implemented globally, including the strategies used for implementation and the facilitators and barriers to achieving desired outcomes. This review will report on a broader set of rounds literature than any previous review by including: (1) any language, (2) quality improvement and other non-research activities, (3) either new interventions or ongoing implementation, and (4) any combination of interprofessional, interdisciplinary, and/or patient-/family-centered rounds. In addition, we will address the scarcity of literature differentiating interprofessional from interdisciplinary rounds. These findings will inform the development and implementation of a team rounds intervention designed for sustainable implementation across resource-variable settings. We also expect to identify gaps in the literature, including countries and regions with limited research on rounding practice and gaps in data along the causal pathway linking specific rounds-related interventions to proximal process indicators and distal outcomes such as patient satisfaction and reduced length of stay. Any gaps identified will inform future research directions.

## Discussion

This systematic review will build upon past reviews by describing reported outcomes of existing literature on inpatient team rounds and by exploring the inclusion of multiple disciplines, in addition to professions and families, across global, multilingual research on rounds. Understanding how care teams come together is particularly important in the care of complex patients, such as those with critical illness, cancer, and cardiovascular disease, where it is common for multiple specialties to be involved in care delivery. Moreover, our review will encompass a range of interventions related to rounds and describe observed effects of these interventions on patient, team, and service outcomes. We will also capture data on both specific implementation outcomes and barriers and facilitators to implementation of rounds reported in the literature.

Traditionally, rounding literature has focused on high-resource, English-speaking settings [6,7,12]. A notable strength of our review is our intentional inclusion of a broad range of studies from global settings by using no language criteria in our search. Through our global network of collaborators in the St. Jude Children’s Research Hospital Global Alliance [28], we are able to identify bilingual reviewers to assist with this approach. In addition, recent literature reviews on rounds have been limited (1) to specific types of team rounds evaluated, such as those that included both multiple professions and patients or families [16], (2) to studies describing only the implementation of a new rounding practice, (3) to implementation in only specific hospital units [7], or (4) to adult patients [6]. This broader landscape review will address these limitations of the existing literature by capturing research describing the diversity of rounding practice across more patient populations and clinical settings to inform a better understanding of how rounding practices are used globally and highlight gaps in existing research.

As in previous literature reviews [12], we may encounter challenges arising from insufficient description of rounding interventions and implementation strategies used. While we are hopeful that recommendations to improve the reporting of interventions and implementation strategies may have improved this [29]; our analysis of specific links between interventions or strategies and outcomes would be limited if these are poorly described. The variety of study designs, interventions, and measures in this review will limit our ability to conduct a meta-analysis to estimate the effectiveness of specific interventions, however this limitation is offset by the robust descriptive and narrative review that represents a broader range of hospital settings and types of interventions.

In conclusion, this systematic review will advance knowledge of how daily team rounds are defined, who they include, what frameworks are used in their development, how they are implemented, and where they are studied across global settings. This will address notable gaps in the existing literature and support efforts to develop interventions to expand the use of team rounds globally to improve communication and coordination of hospital care.

## Supporting information

S1 File S1 search strategy. Search strategy to identify relevant literature in seven databases.
(PDF)

S2 File S2 Data extraction template. Data extraction forms to be completed for each included study.
(PDF)
