# Supplementary material for: Interdisciplinary, interprofessional, patient- and family-centered rounds: A systematic mapping review protocol of global interventions and outcomes
Source: PLoS One. 2026 Oct 6;21(10):e0357968. doi: 10.1371/journal.pone.0357968 (PMC13641390; doi:10.1371/journal.pone.0357968)
Supplement: Supplementary file 1 [file pone.0357968.s001.pdf]

Supplemental File 1: Search Strategy

MERGE Systematic Review Search Strategy and Translation:

Topic: Multidisciplinary Rounds

Date: September 12, 2024

Database (including vendor/platform): MEDLINE (via PubMed)

Set #	
1 Facilities/ inpatient	"Health Facilities"[Mesh] OR "Hospitals"[Mesh] OR "Hospital Units"[Mesh] OR "Academic Medical Centers"[Mesh] OR "Inpatients"[Mesh] OR "Patients' Rooms"[Mesh] OR hospital*[tiab] OR inpatient*[tiab] OR "acute care"[tiab] OR "medical center*"[tiab] OR clinic*[tiab] OR bedside[tiab] OR "bed side"[tiab] OR "in room"[tiab] OR inroom[tiab] OR room[tiab] OR rooms[tiab] OR presence[tiab] OR present[tiab] OR "ward"[tiab] OR "wards"[tiab] OR "care unit*"[tiab]
2 Multidisciplinary	"Patient Care Team"[Mesh] OR "Patient-Centered Care"[Mesh] OR "Interprofessional Relations"[Mesh] OR "Interdisciplinary Communication"[Mesh] OR interprofessional*[tiab] OR inter-professional*[tiab] OR interdisciplinary[tiab] OR inter-disciplinary[tiab] OR intra-disciplinary[tiab] OR intradisciplinary[tiab] OR multidisciplinary[tiab] OR multi-disciplinary[tiab] OR multi-specialty[tiab] OR multispecialty[tiab] OR inter-specialty[tiab] OR interspecialty[tiab] OR sub-specialt*[tiab] OR subspecialt*[tiab] OR "cross professional"[tiab] OR discipline*[tiab] OR disciplinar*[tiab]
3 Rounds	"Teaching Rounds"[Mesh] OR round[tiab] OR rounds[tiab] OR rounding[tiab] OR "morning report*"[tiab]
4	#1 AND #2 AND #3
5	#4 NOT (Editorial[ptyp] OR Letter[ptyp] OR Comment[ptyp] "Case Reports" [ptyp])

Database (including vendor/platform): EMBASE (via Elsevier)

Set #	
1 Facilities/ inpatient	'health care facility'/de OR 'hospital'/de OR 'hospital subdivisions and components'/de OR 'university hospital'/exp OR 'hospital patient'/de OR 'recovery room'/de OR hospital*:ti,ab OR inpatient*:ti,ab OR 'acute care':ti,ab OR 'medical center*':ti,ab OR clinic*:ti,ab OR bedside:ti,ab OR 'bed side':ti,ab OR 'in room':ti,ab OR inroom:ti,ab OR room:ti,ab OR rooms:ti,ab OR presence:ti,ab OR present:ti,ab OR 'ward':ti,ab OR 'wards':ti,ab OR 'care unit*':ti,ab
2 Multidisciplinary	'patient care team'/exp OR 'person centered care'/exp OR 'multidisciplinary team'/exp OR 'interprofessional collaboration'/de OR interprofessional*:ti,ab OR inter-professional*:ti,ab OR interdisciplinary:ti,ab OR inter-disciplinary:ti,ab OR intra-disciplinary:ti,ab OR intradisciplinary:ti,ab OR multidisciplinary:ti,ab OR multi-disciplinary:ti,ab OR multi-specialty:ti,ab OR multispecialty:ti,ab OR inter-specialty:ti,ab OR interspecialty:ti,ab OR sub-specialt*:ti,ab OR

	subspecialt*:ti,ab OR 'cross professional':ti,ab OR discipline*:ti,ab OR disciplinar*:ti,ab
3 Rounds	'teaching round'/exp OR round:ti,ab OR rounds:ti,ab OR rounding:ti,ab OR 'morning report*'
4	#1 AND #2 AND #3
5	#4 AND ([article]/lim OR [article in press]/lim)

Database (including vendor/platform): Cumulative Index of Nursing and Allied Health Literature (CINAHL) (via EBSCO)

Set #	
1 Facilities/ inpatient	(MH "Health Facilities+") OR (MH "Hospitals+") OR (MH "Hospital Units+") OR (MH "Academic Medical Centers") OR (MH "Inpatients") OR (MH "Patients' Rooms+") OR (MH "Patient Bedside") OR TI(hospital* OR inpatient* OR "acute care" OR "medical center*" OR clinic* OR bedside OR "bed side" OR "in room" OR inroom OR room OR rooms OR presence OR present OR "ward" OR "wards" OR "care unit*") OR AB(hospital* OR inpatient* OR "acute care" OR "medical center*" OR clinic* OR bedside OR "bed side" OR "in room" OR inroom OR room OR rooms OR presence OR present OR "ward" OR "wards" OR "care unit*")
2 Multidisciplinary	(MH "Multidisciplinary Care Team+") OR (MH "Patient Centered Care") OR (MH "Interprofessional Relations+") OR TI(interprofessional* OR inter-professional* OR interdisciplinary OR inter-disciplinary OR intra-disciplinary OR intradisciplinary OR multidisciplinary OR multi-disciplinary OR multi-specialty OR multispecialty OR inter-specialty OR interspecialty OR sub-specialt* OR subspecialt* OR "cross professional" OR discipline* OR disciplinar*) OR AB(interprofessional* OR inter-professional* OR interdisciplinary OR inter-disciplinary OR intra-disciplinary OR intradisciplinary OR multidisciplinary OR multi-disciplinary OR multi-specialty OR multispecialty OR inter-specialty OR interspecialty OR sub-specialt* OR subspecialt* OR "cross professional" OR discipline* OR disciplinar*)
3 Rounds	(MH "Patient Rounds") OR TI(round OR rounds OR rounding OR "morning report*") OR AB(round OR rounds OR rounding OR "morning report*")
4	#1 AND #2 AND #3
5	**Limiters** - Publication Type: Clinical Trial, Doctoral Dissertation, Journal Article, Meta Analysis, Meta Synthesis, Randomized Controlled Trial, Systematic Review

Database (including vendor/platform): Web of Science (via Clarivate)

All databases/Exact search: On

Set #	
1 Facilities/ inpatient	TS=(hospital* OR inpatient* OR "acute care" OR "medical center*" OR clinic* OR bedside OR "bed side" OR "in room" OR inroom OR room OR rooms OR presence OR present OR "ward" OR "wards" OR "care unit*")
2 Multidisciplinary	TS=(interprofessional* OR inter-professional* OR interdisciplinary OR inter-disciplinary OR intra-disciplinary OR intradisciplinary OR multidisciplinary OR

	multi-disciplinary OR multi-specialty OR multispecialty OR inter-specialty OR interspecialty OR sub-specialt* OR subspecialt* OR "cross professional" OR discipline* OR disciplinar*)
3 Rounds	TS=(round OR rounds OR rounding OR "morning report*")
4	#1 AND #2 AND #3
5	#4 Limit to Document Types: Article, Dissertation Thesis, Review Article, and Clinical Trial

Database (including vendor/platform): Global Health (via CABI)

Set #	
1 Facilities/ inpatient	ab:(hospital* OR inpatient* OR "acute care" OR "medical center*" OR clinic* OR bedside OR "bed side" OR "in room" OR inroom OR room OR rooms OR presence OR present OR "ward" OR "wards" OR "care unit*") OR Title:(hospital* OR inpatient* OR "acute care" OR "medical center*" OR clinic* OR bedside OR "bed side" OR "in room" OR inroom OR room OR rooms OR presence OR present OR "ward" OR "wards" OR "care unit*")
2 Multidisciplinary	ab:(interprofessional* OR inter-professional* OR interdisciplinary OR inter-disciplinary OR intra-disciplinary OR intradisciplinary OR multidisciplinary OR multi-disciplinary OR multi-specialty OR multispecialty OR inter-specialty OR interspecialty OR sub-specialt* OR subspecialt* OR "cross professional" OR discipline* OR disciplinar*) OR Title:(interprofessional* OR inter-professional* OR interdisciplinary OR inter-disciplinary OR intra-disciplinary OR intradisciplinary OR multidisciplinary OR multi-disciplinary OR multi-specialty OR multispecialty OR inter-specialty OR interspecialty OR sub-specialt* OR subspecialt* OR "cross professional" OR discipline* OR disciplinar*)
3 Rounds	ab:(round OR rounds OR rounding OR "morning report*") OR Title:(round OR rounds OR rounding OR "morning report*")
4	#1 AND #2 AND #3
5	#4 Limit to Document Types: Journal Article

Database (including vendor/platform): ERIC (via ed.gov)

Set #	
1	(patient* OR hospital* OR "care unit" OR "care units") AND round*
2	Limit to Journal Articles

Database (including vendor/platform): Social Sciences Full Text (H.W. Wilson) (via EBSCO)

Set #	
1 Facilities/ inpatient	TI (hospital* OR inpatient* OR "acute care" OR "medical center*" OR clinic* OR bedside OR "bed side" OR "in room" OR inroom OR room OR rooms OR presence OR present OR "ward" OR "wards" OR "care unit*") OR AB (hospital* OR inpatient* OR "acute care" OR "medical center*" OR clinic* OR bedside OR "bed side" OR "in room" OR inroom OR room OR rooms OR presence OR present OR "ward" OR "wards" OR "care unit*")

2 Multidisciplinary	TI (interprofessional* OR inter-professional* OR interdisciplinary OR inter-disciplinary OR intra-disciplinary OR intradisciplinary OR multidisciplinary OR multi-disciplinary OR multi-specialty OR multispecialty OR inter-specialty OR interspecialty OR sub-specialt* OR subspecialt* OR "cross professional" OR discipline* OR disciplinar*) OR AB (interprofessional* OR inter-professional* OR interdisciplinary OR inter-disciplinary OR intra-disciplinary OR intradisciplinary OR multidisciplinary OR multi-disciplinary OR multi-specialty OR multispecialty OR inter-specialty OR interspecialty OR sub-specialt* OR subspecialt* OR "cross professional" OR discipline* OR disciplinar*)
3 Rounds	TI (round OR rounds OR rounding OR "morning report*") OR AB (round OR rounds OR rounding OR "morning report*")
4	#1 AND #2 AND #3
5	**Limiters** - Document Type: Article, Review Article
