# Supplementary material for: Interdisciplinary, interprofessional, patient- and family-centered rounds: A systematic mapping review protocol of global interventions and outcomes
Source: PLoS One. 2026 Oct 6;21(10):e0357968. doi: 10.1371/journal.pone.0357968 (PMC13641390; doi:10.1371/journal.pone.0357968)
Supplement: Supplementary file 2 [file pone.0357968.s002.pdf]

General Study Characteristics

Record ID ______________________________

Study Characteristics

Covidence ID ______________________________

Authors ______________________________

Publication year ______________________________

Title ______________________________

Journal ______________________________

Name of first reviewer: ______________________________

Name of second reviewer: ______________________________

Upload Documents

doi (include https://doi.org/) ____________________

Article
obtain from Covidence or article link

Supplement

If you are unable to access supplements or there are more than 2 supplemental files, send a data query to the current MERGE Coordinator.

Additional Supplement

General Study Details

Should the article be excluded from this review?

- ◯ Include
- ◯ Exclude

Why should It be excluded?

- ◯ Wrong study type
- ◯ Wrong study setting
- ◯ Not daily rounds on patient care
- ◯ Not multidisciplinary, multiprofessional, or family-centered
- ◯ No assessment or outcome
- ◯ Duplicate study

You have indicated that this article should be excluded. If this is correct, please also note this on our "Tracking Spreadsheet". There is no need to complete this form further.

In what country or countries did the study occur? (choose all)

- ☐ Afghanistan
- ☐ Albania
- ☐ Algeria
- ☐ Andorra
- ☐ Angola
- ☐ Antigua & Barbuda
- ☐ Argentina
- ☐ Armenia
- ☐ Australia
- ☐ Austria
- ☐ Azerbaijan
- ☐ Bahamas
- ☐ Bahrain
- ☐ Bangladesh
- ☐ Barbados
- ☐ Belarus
- ☐ Belgium
- ☐ Belize
- ☐ Benin
- ☐ Bhutan
- ☐ Bolivia (Plurinational State of)
- ☐ Bosnia and Herzegovina
- ☐ Botswana
- ☐ Brazil
- ☐ Brunei Darussalam
- ☐ Bulgaria
- ☐ Burkina Faso
- ☐ Burundi
- ☐ Cabo Verde
- ☐ Cambodia
- ☐ Cameroon
- ☐ Canada
- ☐ Central African Republic
- ☐ Chad
- ☐ Chile
- ☐ China
- ☐ Colombia
- ☐ Comoros
- ☐ Congo
- ☐ Congo, Democratic Republic of the
- ☐ Cook Islands
- ☐ Costa Rica
- ☐ Cote d'Ivoire
- ☐ Croatia
- ☐ Cuba
- ☐ Cyprus
- ☐ Czechia
- ☐ Denmark
- ☐ Djibouti
- ☐ Dominica
- ☐ Dominican Republic
- ☐ Ecuador
- ☐ Egypt
- ☐ El Salvador
- ☐ Equatorial Guinea
- ☐ Eritrea
- ☐ Estonia
- ☐ Eswatini
- ☐ Ethiopia
- ☐ Fiji
- ☐ Finland
- ☐ France
- ☐ Gabon
- ☐ Gambia
- ☐ Georgia
- ☐ Germany
- ☐ Ghana
- ☐ Greece
- ☐ Grenada
- ☐ Guatemala
- ☐ Guinea
- ☐ Guinea-Bissau
- ☐ Guyana
- ☐ Haiti
- ☐ Holy See
- ☐ Honduras
- ☐ Hungary
- ☐ Iceland
- ☐ India
- ☐ Indonesia
- ☐ Iran (Islamic Republic of)
- ☐ Iraq
- ☐ Ireland
- ☐ Israel
- ☐ Italy
- ☐ Jamaica
- ☐ Japan
- ☐ Jordan
- ☐ Kazakhstan
- ☐ Kenya
- ☐ Kiribati
- ☐ Korea, Democratic People's Republic of
- ☐ Korea, Republic of
- ☐ Kuwait
- ☐ Kyrgyzstan
- ☐ Lao People's Democratic Republic
- ☐ Latvia
- ☐ Lebanon
- ☐ Lesotho
- ☐ Liberia
- ☐ Libya
- ☐ Liechtenstein
- ☐ Lithuania
- ☐ Luxembourg
- ☐ Madagascar
- ☐ Malawi
- ☐ Malaysia
- ☐ Maldives
- ☐ Mali
- ☐ Malta
- ☐ Marshall Islands
- ☐ Mauritania
- ☐ Mauritius
- ☐ Mexico
- ☐ Micronesia (Federated States of)
- ☐ Moldova, Republic of
- ☐ Monaco
- ☐ Mongolia
- ☐ Montenegro
- ☐ Morocco
- ☐ Mozambique
- ☐ Myanmar
- ☐ Namibia
- ☐ Nauru
- ☐ Nepal
- ☐ Netherlands (Kingdom of the)
- ☐ New Zealand
- ☐ Nicaragua
- ☐ Niger
- ☐ Nigeria
- ☐ Niue
- ☐ North Macedonia
- ☐ Norway
- ☐ Oman
- ☐ Pakistan
- ☐ Palau
- ☐ Panama
- ☐ Palestine, State of
- ☐ Papua New Guinea
- ☐ Paraguay
- ☐ Peru
- ☐ Philippines
- ☐ Poland
- ☐ Portugal
- ☐ Qatar
- ☐ Romania
- ☐ Russian Federation
- ☐ Rwanda
- ☐ Saint Kitts and Nevis
- ☐ Saint Lucia
- ☐ Saint Vincent and the Grenadines
- ☐ Samoa
- ☐ San Marino
- ☐ Sao Tome and Principe
- ☐ Saudi Arabia
- ☐ Senegal
- ☐ Serbia
- ☐ Seychelles
- ☐ Sierra Leone
- ☐ Singapore
- ☐ Slovakia
- ☐ Slovenia
- ☐ Solomon Islands
- ☐ Somalia
- ☐ South Africa
- ☐ South Sudan
- ☐ Spain
- ☐ Sri Lanka
- ☐ Sudan
- ☐ Suriname
- ☐ Sweden
- ☐ Switzerland
- ☐ Syrian Arab Republic
- ☐ Taiwan
- ☐ Tajikistan
- ☐ Tanzania, United Republic of
- ☐ Thailand
- ☐ Timor-Leste
- ☐ Togo
- ☐ Tonga
- ☐ Trinidad and Tobago
- ☐ Tunisia
- ☐ Turkey
- ☐ Turkmenistan
- ☐ Tuvalu
- ☐ Uganda
- ☐ Ukraine
- ☐ United Arab Emirates
- ☐ United Kingdom of Great Britain and Northern Ireland
- ☐ United States of America
- ☐ Uruguay
- ☐ Uzbekistan
- ☐ Vanuatu
- ☐ Venezuela, Bolivarian Republic of
- ☐ Vietnam
- ☐ Yemen
- ☐ Zambia
- ☐ Zimbabwe
- ☐ Unclear/not described
- ☐ Other, specify _______

In what language is the study?	☐ English<br>☐ Arabic<br>☐ German<br>☐ Italian<br>☐ Korean<br>☐ Norwegian<br>☐ Portuguese<br>☐ Spanish<br>☐ Swedish<br>☐ Other, specify ______
How was the study funded: (check all that apply)	☐ Government (including FDA, NIH, CDC, CIHR, etc)<br>☐ Non-governmental organization (NGO), Including Scientific Societies, Hospital Foundation, etc<br>☐ Industry<br>☐ None<br>☐ Unclear/not described<br>☐ Other (please describe)
Other, specify:	__________________________________

Hospital Characteristics

Did the study occur in a single center or multiple centers?	◯ Single center<br>◯ Multiple centers<br>◯ Unclear/not described
Type of hospital:	◯ General hospital (adult and pediatric)<br>◯ Pediatric hospital<br>◯ Adult hospital<br>◯ Specialty hospital (e.g. oncology, orthopedic)<br>◯ Other (please describe)<br>◯ Unclear/not described
Other, specify:	__________________________________
Is the hospital a teaching hospital (academic)?	◯ Teaching/academic hospital<br>◯ Not a teaching/academic hospital<br>◯ Unclear/not described
What is the referral level of the hospital?	◯ Community/primary hospital<br>◯ Tertiary/referral hospital<br>◯ Unclear/not described
How many centers:	____________________________

Patient/Unit Characteristics

What inpatient setting was the study conducted (pick all that apply)?

- ☐ Inpatient ward
- ☐ Step-down/Intermediate care unit
- ☐ Intensive care unit
- ☐ Other (please describe)
- ☐ Unclear/not described

Other, specify:

____

What is the age group of the patient population?

- ◯ Adult
- ◯ Pediatric
- ◯ Mixed
- ◯ Unclear/not described

Does the study focus on patients with a specific medical condition?

- ◯ Yes
- ◯ No
- ◯ Unclear/not described

Which patient population (select all that apply):

- ☐ Oncology
- ☐ Cardiology
- ☐ Neurology
- ☐ Surgery
- ☐ Medical/Surgical
- ☐ Other, please describe:

Other, specify:

____

Study Methods

Study methodology:

- ◯ Qualitative
- ◯ Quantitative
- ◯ Mixed-methods (quant and qual data are integrated)
- ◯ Multiple methods (quant and qual data, but not integrated)
- ◯ Unclear/not described

Quantitative study methodology:

randomized controlled trial participants are allocated to intervention or control groups by randomization

non-randomized study any quantitative studies estimating the effectiveness of an intervention or studying other exposures that do not use randomization to allocate units to comparison groups (cohort, case-control, pre-post)

descriptive study designed only to describe the existing distribution of variables without much regard to causal relationships or other hypotheses

- ◯ Randomized control trial
- ◯ Non-randomized study
- ◯ Descriptive
- ◯ Unclear/not described

Was the study design described using any of these terms?
Check all that apply

- ☐ Phenomenology
- ☐ Grounded Theory
- ☐ Ethnography
- ☐ Participatory Research
- ☐ Cluster-randomized controlled trial
- ☐ Hybrid (effectiveness-implementation) trial
- ☐ Cross-sectional Study
- ☐ Cohort Study
- ☐ Case-Control Study
- ☐ Pre-Post (Before/After) Study
- ☐ Time Series
- ☐ Explanatory sequential (QUAN-QUAL) Mixed Methods
- ☐ Exploratory sequential (QUAL-QUAN) Mixed Methods
- ☐ Convergent (Parallel) Mixed Methods
- ☐ Quality Improvement
- ☐ None of the above

Copy and paste the study design from the text.

____

Does this study design make any comparisons?
This may be across sites, before/after, or any other type of comparison.

- ◯ Yes
- ◯ No
- ◯ Unclear/not described

For any comparisons reported, briefly describe what or who was compared.
For example, indicate if comparison groups were based on intervention, site, timepoint, etc.

____

What best describes the objective(s) of this study: (check all that apply, can be quantitative or qualitative)	☐ Develop or implement a new team rounding process, change in team rounding process (e.g. addition of a new team member), rounding support tool (e.g. checklist), and/or strategies to support implementation<br>☐ Assess the association between rounds or a rounding intervention on an outcome of interest (including implementation, clinical, service, and educational outcomes)<br>☐ Assess determinants of rounds implementation success or failure (barriers and facilitators)<br>☐ Main objective of the study is not related to rounds (rounds are part multicomponent "parent" intervention, rounds is the setting to study something else)<br>☐ Other (please describe)
Other, specify:	__________________________________
What data sources were used in this study (check all that apply)?	☐ Clinical (registry or medical record) data<br>☐ Surveys<br>☐ Interviews or focus groups<br>☐ Observations<br>☐ Hospital policies or manuals<br>☐ Other (please describe)
Other, specify:	__________________________________
Is a case report form or data collection instrument provided in the paper or supplemental materials?	◯ Yes<br>◯ No
Please describe the survey that was used:<br>Include relevant info on the following: goal, question types (likert, text response), number of questions, topics covered, validated survey scales	__________________________________
Is a copy of the survey provided in the paper or supplemental materials?	◯ Yes<br>◯ No
Is a focus group or interview guide provided in the paper or supplemental materials?	◯ Yes<br>◯ No
Is a qualitative code book provided in the paper or supplemental materials?	◯ Yes<br>◯ No
Is an observation guide or tool provided in the paper or supplemental materials (or describe in enough detail to receate it)?	◯ Yes<br>◯ No

For the surveys, interviews, observations and/or "other" data sources:
What types of individuals participated (choose all that apply):

- ☐ Patient
- ☐ Family member
- ☐ Physician
- ☐ Advanced practice provider (NP or PA)
- ☐ Bedside nurse
- ☐ Pharmacist
- ☐ Respiratory therapist
- ☐ Social worker
- ☐ Case manager
- ☐ Charge nurse
- ☐ Other (please describe)
- ☐ Unclear/not described

Other, specify: ________________________________

Sample Size:
Indicate the sample size of the largest data collection activity.
If not applicable, enter negative 99 (-99). __________________________

Description Of Rounds

Terminology used to describe rounds (check all that apply):

- ☐ Multidisciplinary
- ☐ Interdisciplinary
- ☐ Multiprofessional
- ☐ Interprofessional
- ☐ Family-centered
- ☐ Family-inclusive
- ☐ Other: (please describe)
- ☐ Unclear/not described

Other, specify: ______________________________

The next questions are about who is included in rounds. If the individuals included in rounds changed over time or between sites, answer based on the most inclusive version of rounds described.

Described team composition on rounds (profession): (check all that apply)

- ☐ Physician
- ☐ Advanced practice provider (NP or PA)
- ☐ Nurse (bedside or unspecified)
- ☐ Pharmacist
- ☐ Respiratory therapist
- ☐ Social worker
- ☐ Patient
- ☐ Family member
- ☐ Case manager
- ☐ Charge nurse
- ☐ Dietician/Nutritionist
- ☐ Other (please describe)
- ☐ Unclear/not described

Other, specify: ______________________________

Are trainees (of any profession) present on rounds?

- ◯ Yes
- ◯ No
- ◯ Unclear/not described

What types of trainees (check all that apply)

- ☐ Medical student
- ☐ Resident or Fellow (physician trainee)
- ☐ Advanced practice provider (NP or PA) trainee
- ☐ Nursing trainees
- ☐ Pharmacist trainees
- ☐ Respiratory therapist trainees
- ☐ Social worker trainees
- ☐ Other (please describe)

Other, describe: ______________________________

Described team composition on rounds (disciplines):<br>(check all that apply)	☐ General Medicine (including Hospitalist)<br>☐ Oncology<br>☐ Surgery (any specialty)<br>☐ Palliative Care<br>☐ Intensive Care<br>☐ Emergency Medicine<br>☐ Cardiology<br>☐ Neurology<br>☐ Nephrology<br>☐ Pulmonology<br>☐ Psychiatry<br>☐ Endocrinology<br>☐ Hematology<br>☐ Pediatric Medicine (general or sub-specialty)<br>☐ Consultants for the patient (specific type(s) not described)<br>☐ Other (please describe)<br>☐ Unclear/not described
Pediatric Medicine, specify:	☐ General Pediatric (including Hospitalist)<br>☐ Pediatric Oncology<br>☐ Pediatric Surgery<br>☐ Pediatric Palliative Care<br>☐ Pediatric Intensive Care<br>☐ Pediatric Emergency Medicine<br>☐ Pediatric Cardiology<br>☐ Pediatric Neurology<br>☐ Pediatric Nephrology<br>☐ Pediatric Pulmonology<br>☐ Pediatric Psychiatric<br>☐ Pediatric Endocrinology<br>☐ Pediatric Hematology<br>(If "other" is needed, use the option in the previous question)
Other, specify:	__________________________________
Does more than one discipline round together?<br><br>Examples: Intensive care and Surgery; Pediatrician and Consultant, etc.	◯ Yes<br>◯ No<br>◯ Unclear/not described
Is the team composition (profession, discipline, trainee) described or compared across centers?	◯ Yes<br>◯ No
If yes, how many?:	____________________________
Is a change or variation in the team composition during rounds described? (patient/family, professions, disciplines, or trainees)	◯ Yes<br>◯ No<br>◯ Unclear
What aspect of the team composition of rounds changed?	☐ Patient/Family<br>☐ Professions<br>☐ Trainees<br>☐ Disciplines

Which professions experienced a change in their inclusion in rounds?	☐ Physician<br>☐ Advanced practice provider (NP or PA)<br>☐ Bedside nurse<br>☐ Pharmacist<br>☐ Respiratory therapist<br>☐ Social worker<br>☐ Case manager<br>☐ Charge nurse<br>☐ Dietician/Nutritionist<br>☐ Other (please describe)______<br>☐ Unclear/not described
Which trainees experienced a change in their inclusion in rounds?	☐ Medical student<br>☐ Resident, Intern or Fellow (physician trainee)<br>☐ Advanced practice provider (NP or PA) trainee<br>☐ Nursing trainees<br>☐ Pharmacist trainees<br>☐ Respiratory therapist trainees<br>☐ Social worker trainees<br>☐ Other (please describe)
Which disciplines experienced a change in their inclusion in rounds?	☐ General Medicine<br>☐ Oncology<br>☐ Surgery (any specialty)<br>☐ Palliative Care<br>☐ Intensive Care<br>☐ Emergency Medicine<br>☐ Cardiology<br>☐ Neurology<br>☐ Nephrology<br>☐ Pulmonology<br>☐ Psychiatry<br>☐ Endocrinology<br>☐ Hematology<br>☐ Pediatric Medicine (general or sub-specialty)<br>☐ Other (please describe)<br>☐ Unclear/not described
Pediatric medicine, specify	☐ General Pediatric<br>☐ Pediatric Oncology<br>☐ Pediatric Surgery<br>☐ Pediatric Palliative Care<br>☐ Pediatric Intensive Care<br>☐ Pediatric Emergency Medicine<br>☐ Pediatric Cardiology<br>☐ Pediatric Neurology<br>☐ Pediatric Nephrology<br>☐ Pediatric Pulmonology<br>☐ Pediatric Psychiatric<br>☐ Pediatric Endocrinology<br>☐ Pediatric Hematology
Other, specify:	__________________________________
Where physically are rounds conducted? (select all that apply)	☐ Hallway or Nurses Station (outside of patient room)<br>☐ Bedside (inside of patient room)<br>☐ Conference or team room<br>☐ Virtual<br>☐ Unclear/not described

Did the study use an existing rounding framework or propose/develop a new rounding framework? (select all that apply)	☐ PFC-IPASS (Patient & family-centered - IPASS)<br>☐ RAMPED-UP (Respiratory, Activity, Medication , Pain & Palliative measures Psychological data, Enteral feeding , Disposition , Updates and questions, Prophylaxis)<br>☐ SIBR (Structured Interdisciplinary Bedside Rounds)<br>☐ Other existing framework (please describe)<br>☐ Developed new framework (please describe)<br>☐ No rounds framework<br>☐ Unclear
Other, specify:	__________________________________
New, specify:	__________________________________
Did the study develop, implement, or evaluate an intervention or implementation strategy related to rounds?<br><br>(This may be a new intervention or evaluation of past implementation)	◯ Yes<br>◯ No<br>◯ Unclear/not described
Did the intervention include a new rounding process or rounding support tool (e.g. checklist)?	◯ New rounding process<br>◯ Rounding support tool (e.g. checklist)<br>◯ Both<br>◯ Other<br>◯ Unclear/not described
Please describe the intervention, process, or tool being implemented:	__________________________________
Was this rounds intervention part of a multi-component parent intervention that included activities unrelated to rounds?	◯ Yes<br>◯ No<br>◯ Unclear/not described
Please describe the multi-component parent intervetion	__________________________________
Why was a change in the rounding process being proposed (what's the knowledge gap or problem)?	__________________________________
What aspect of rounds was the intervention trying to improve or change? (check all that apply)	☐ Rounding time/duration<br>☐ Rounding process standardization (complete all elements of a defined process)<br>☐ Inclusion (participation of healthcare providers and/or family member)<br>☐ Satisfaction (satisfaction of healthcare providers and/or family members with rounds)<br>☐ Location of rounds<br>☐ Other (please describe)<br>☐ Unclear/not described

Other, specify: __________________________________

Were any of the following structural/organizational characteristics described as part of the new rounding process? (check all that apply)

- ☐ Establishing a start time for daily rounds (i.e. 9am)
- ☐ Determining patient rounding order prior to starting rounds
- ☐ Communicating with bedside nurse (i.e. calling prior to their patient)
- ☐ Physical arrangement of rounds (i.e. where to stand)
- ☐ Structure/Process to guide rounding process
- ☐ Specific roles for rounding participants (i.e. care team members)
- ☐ Intentional engagement with patient/families
- ☐ How to interact with interpreters for patients with different language fluency
- ☐ Designation of who would enter orders during rounds
- ☐ Checklist reviewed
- ☐ Clinical pathway status reviewed
- ☐ Plan of care verbally summarized at end of rounds
- ☐ Plan of care summarized visually during or at end of rounds (i.e. on white board)
- ☐ Other characteristic (please describe)
- ☐ None of the above were mentioned

Other, specify: __________________________________

Which implementation strategies were used to support the implementation of rounds or support tool? (choose all that apply)

- ☐ Assessing potential implementation barriers/enablers (determinants)
- ☐ Planning implementation activities (implementation strategizing)
- ☐ Training and education of clinical staff
- ☐ Adaptation of the rounding intervention or tool to local context
- ☐ Engaging stakeholders (clinicians, hospital leaders)
- ☐ Audit and feedback on how implementation was going
- ☐ External facilitation (coaching) from someone outside the institution/unit
- ☐ Local (internal) implementation champions
- ☐ Incentives to clinical staff
- ☐ Handouts or visual guides (e.g. physical lists, cards)
- ☐ Other (please describe)
- ☐ Unclear/not described

Other, specify: __________________________________

Study Findings

What types of outcomes are assessed in this study?

- ☐ Implementation outcomes (e.g. acceptability, feasibility, appropriateness, etc.)
- ☐ Implementation barriers and facilitators
- ☐ Rounds-specific outcomes (duration of rounds, participation in rounds, etc)
- ☐ Patient outcomes (e.g. mortality, length-of-stay, satisfaction)
- ☐ Family outcome (e.g. stress, satisfaction with care)
- ☐ Healthcare provider outcomes (e.g. perspectives, intent to leave, burn out, wellbeing)
- ☐ Team outcomes (e.g. communication quality, teamwork)
- ☐ Service outcomes (e.g. safety events, medical errors, hospital-acquired infections)
- ☐ Educational outcomes (learning for trainees)
- ☐ Other (please describe)
- ☐ None of the above

(outcome assessment may be quantitative or qualitative, choose all that apply,)

You indicated that there are no outcomes reported. This is possible if the article is reporting the development of a new rounds intervention, but otherwise may indicate that the article should be excluded.
If there are outcomes not listed here, choose "Other" instead.

Other, specify:

____

In this multicomponent intervention, are the outcomes reported specific to the rounds component?

- ◯ Yes, outcome are specific to rounds component
- ◯ No, outcomes are reported for the whole parent intervention
- ◯ Mixed, Some outcomes for parent intervention, some specific to rounds
- ◯ Unclear/not described

Implementation Outcomes

Implementation Outcomes How is this outcome assessed? Specific outcome Is there an assessment of change, and what did it show?
Acceptability

The perception among implementation stakeholders that an innovation is agreeable, palatable, or satisfactory

______ ______ ______

Adoption The intention, initial decision, or action to try or employ an innovation or evidence-based practice

______ ______ ______

Appropriateness The perceived fit, relevance, or compatibility of the innovation or evidence-based practice for a given practice setting; and/or perceived fit of the innovation to address a particular issue or problem

______ ______ ______

Cost The cost impact of an implementation effort

______ ______ ______

Feasability The extent to which a new treatment, or an innovation, can be successfully used or carried out within a given agency or setting

______ ______ ______

Fidelity The degree to which an intervention was implemented as it was prescribed in the original protocol or as it was intended by the program developers

______ ______ ______

Penetration The integration of a practice within a service setting and its subsystems

______ ______ ______

Sustainability The extent to which a newly implemented treatment is maintained or institutionalized within a service setting's ongoing, stable operations

______ ______ ______

Other Implementation Outcomes ______ ______ ______

Acceptability	
Summarize acceptability findings.	__________________________________
Copy and paste main acceptability findings from text.	__________________________________
Was the change in acceptability statistically significant?<br>If there are multiple outcomes, respond "yes" if at least one is siginificant.	◯ Yes<br>◯ No<br>◯ Significance testing not reported<br>◯ Unclear/not described
Adoption	
Summarize adoption findings.	__________________________________
Copy and paste main adoption findings from text.	__________________________________
Was the change in adoption statistically significant?<br>If there are multiple outcomes, respond "yes" if at least one is siginificant.	◯ Yes<br>◯ No<br>◯ Significance testing not reported<br>◯ Unclear/not described
Appropriateness	
Summarize appropriateness findings.	__________________________________
Copy and paste main appropriateness findings from text.	__________________________________
Was the change in appropriateness statistically significant?<br>If there are multiple outcomes, respond "yes" if at least one is siginificant.	◯ Yes<br>◯ No<br>◯ Significance testing not reported<br>◯ Unclear/not described

Cost

Summarize cost findings.

____

Copy and paste main cost findings from text.

____

Was the change in cost statistically significant?
If there are multiple outcomes, respond "yes" if at least one is siginificant.

- ◯ Yes
- ◯ No
- ◯ Significance testing not reported
- ◯ Unclear/not described

Feasability

Summarize feasibility findings.

____

Copy and paste main feasibility findings from text.

____

Was the change in feasibility statistically significant?
If there are multiple outcomes, respond "yes" if at least one is siginificant.

- ◯ Yes
- ◯ No
- ◯ Significance testing not reported
- ◯ Unclear/not described

Fidelity

Summarize fidelity findings.

____

Copy and paste main fidelity findings from text.

____

Was the change in fidelity statistically significant?
If there are multiple outcomes, respond "yes" if at least one is siginificant.

- ◯ Yes
- ◯ No
- ◯ Significance testing not reported
- ◯ Unclear/not described

Penetration

Summarize penetration findings.

____

Copy and paste main penetration findings from text.

____

Was the change in penetration statistically significant?
If there are multiple outcomes, respond "yes" if at least one is siginificant.

- ◯ Yes
- ◯ No
- ◯ Significance testing not reported
- ◯ Unclear/not described

Sustianability

Summarize sustainability findings.

____

Copy and paste main sustainability findings from text.

____

Was the change in sustainability statistically significant?
If there are multiple outcomes, respond "yes" if at least one is siginificant.

- ◯ Yes
- ◯ No
- ◯ Significance testing not reported
- ◯ Unclear/not described

Other Implementation Outcomes

Summarize findings of the other implementation outcomes.

____

Copy and paste main findings about these other implementation outcomes from text.

____

Was the change in these other implementation outcomes statistically significant?
If there are multiple outcomes, respond "yes" if at least one is siginificant.

- ◯ Yes
- ◯ No
- ◯ Significance testing not reported
- ◯ Unclear/not described

Implementation Barriers and Facilitators

Barriers and facilitators were assessed in relation to:

- ◯ Current rounds practice (no change introduced)
- ◯ An upcoming change to rounds (hypothetical)
- ◯ Following implementation of a change to rounds
- ◯ Other (please describe)
- ◯ Unclear/not described

Which framework was used to describe determinants of rounding implementation success or failure?

- ◯ Consolidated Framework for Implementation Research (CFIR)
- ◯ PRISM
- ◯ "Theoretical Domain Framework (TDF) or Capability, Opportunity, Motivation - Behavior (COMB)"
- ◯ EPIS
- ◯ Organizational Readiness for Implementing Change (ORIC)
- ◯ Other (please describe)
- ◯ Unclear/not described

Other, specify:

____

Summarize identified facilitators of implementing rounds (or a rounds intervention): ______________________________

Summarize identified barriers of implementing rounds (or a rounds intervention): ______________________________

Summarize any additional important findings related to barriers and facilitators of rounds (or a rounds intervention): ______________________________

Rounds-specific Outcomes

Rounds outcomes How is this outcome assessed? Specific outcome Is there an assessment of change, and what did it show?

Rounding time/duration

______ ______ ______

Rounding process standardization

______ ______ ______

Inclusion of health care team members

______ ______ ______

Inclusion of patient/families

______ ______ ______

Other rounds-specific measures

______ ______ ______

Rounding time/duration

Summarize rounding time/duration findings. ______________________________

Copy and paste main rounding time/duration findings from text. ______________________________

Was the change in rounding time/duration statistically significant?
If there are multiple outcomes, respond "yes" if at least one is siginificant.

- ◯ Yes
- ◯ No
- ◯ Significance testing not reported
- ◯ Unclear/not described

Rounds standardized process

Summarize rounds standardized process findings.

____

Copy and paste main rounds standardized process findings from text.

____

Was the change in rounds standardized process statistically significant?
If there are multiple outcomes, respond "yes" if at least one is siginificant.

- ◯ Yes
- ◯ No
- ◯ Significance testing not reported
- ◯ Unclear/not described

Inclusion of health care team members on rounds

Summarize inclusion of health care team members on rounds findings.

____

Copy and paste main inclusion of health care team members on rounds findings from text.

____

Was the change in inclusion of health care team members on rounds statistically significant?
If there are multiple outcomes, respond "yes" if at least one is siginificant.

- ◯ Yes
- ◯ No
- ◯ Significance testing not reported
- ◯ Unclear/not described

Inclusion of patients/families on rounds

Summarize inclusion of patients/families on rounds findings.

____

Copy and paste main inclusion of patients/families on rounds findings from text.

____

Was the change in inclusion of patients/families on rounds statistically significant?
If there are multiple outcomes, respond "yes" if at least one is siginificant.

- ◯ Yes
- ◯ No
- ◯ Significance testing not reported
- ◯ Unclear/not described

Other rounds-specific measure

Summarize other rounds-specific measure findings.

____

Copy and paste main other rounds-specific measure findings from text.

____

Was the change in other rounds-specific measure statistically significant?
If there are multiple outcomes, respond "yes" if at least one is siginificant.

- ◯ Yes
- ◯ No
- ◯ Significance testing not reported
- ◯ Unclear/not described

Patient Outcomes

Patient Outcomes How is this outcome assessed? Specific outcome Is there an assessment of change, and what did it show?

Patient Mortality

______ ______ ______

Length of Stay

______ ______ ______

Patient Satisfaction

______ ______ ______

Other Patient Outcome

______ ______ ______

Patient Mortality

Summarize mortality findings. ________________________________

Copy and paste main mortality findings from text. ________________________________

Was the change in mortality statistically significant? If there are multiple outcomes, respond "yes" if at least one is siginificant.
- ◯ Yes
- ◯ No
- ◯ Significance testing not reported
- ◯ Unclear/not described

Length of stay

Summarize length of stay findings. ________________________________

Copy and paste main length of stay findings from text. ________________________________

Was the change in length of stay statistically significant? If there are multiple outcomes, respond "yes" if at least one is siginificant.
- ◯ Yes
- ◯ No
- ◯ Significance testing not reported
- ◯ Unclear/not described

Patient statisfaction

Summarize patient statisfaction findings.

____

Copy and paste main patient statisfaction findings from text.

____

Was the change in patient statisfaction statistically significant?
If there are multiple outcomes, respond "yes" if at least one is siginificant.

- ◯ Yes
- ◯ No
- ◯ Significance testing not reported
- ◯ Unclear/not described

Other patient Outcomes

Summarize findings of the other patient outcomes.

____

Copy and paste main findings about these other patient outcomes from the text.

____

Was the change in these other patient outcomes statistically significant?
If there are multiple outcomes, respond "yes" if at least one is siginificant.

- ◯ Yes
- ◯ No
- ◯ Significance testing not reported
- ◯ Unclear/not described

Family Outcomes

Family Outcomes How is this outcome assessed? Specific outcome Is there an assessment of change, and what did it show?

Family stress

______ ______ ______

Family knowledge

______ ______ ______

Satisfaction with care

______ ______ ______

Other family outcomes

______ ______ ______

Family stress

Summarize family stress findings.

____

Copy and paste main family stress findings from text.

____

Was the change in family stress statistically significant?
If there are multiple outcomes, respond "yes" if at least one is siginificant.

- ◯ Yes
- ◯ No
- ◯ Significance testing not reported
- ◯ Unclear/not described

Family knowledge

Summarize family knowledge findings.

____

Copy and paste main family knowledge findings from text.

____

Was the change in family knowledge statistically significant?
If there are multiple outcomes, respond "yes" if at least one is siginificant.

- ◯ Yes
- ◯ No
- ◯ Significance testing not reported
- ◯ Unclear/not described

Satisfaction with care

Summarize satisfaction with care findings.

____

Copy and paste main satisfaction with care findings from text.

____

Was the change in satisfaction with care statistically significant?
If there are multiple outcomes, respond "yes" if at least one is siginificant.

- ◯ Yes
- ◯ No
- ◯ Significance testing not reported
- ◯ Unclear/not described

Other family outcomes

Summarize other family outcomes findings.

____

Copy and paste main other family outcomes findings from text.

____

Was the change in other family outcomes statistically significant?
If there are multiple outcomes, respond "yes" if at least one is siginificant.

- ◯ Yes
- ◯ No
- ◯ Significance testing not reported
- ◯ Unclear/not described

Health Worker Outcomes

Health Worker Outcomes How is this outcome assessed? Specific outcome Is there an assessment of change, and what did it show?

Burnout

______ ______ ______

Intent to leave

______ ______ ______

Job Satisfaction

______ ______ ______

Workload

______ ______ ______

Other health worker outcome

______ ______ ______

Burnout

Summarize burnout findings. ________________________________

Copy and paste main burnout findings from text. ________________________________

Was the change in burnout statistically significant?
If there are multiple outcomes, respond "yes" if at least one is siginificant.

- ○ Yes
- ○ No
- ○ Significance testing not reported
- ○ Unclear/not described

Intent to leave

Summarize intent to leave findings. ________________________________

Copy and paste main intent to leave findings from text. ________________________________

Was the change in intent to leave statistically significant?
If there are multiple outcomes, respond "yes" if at least one is siginificant.

- ○ Yes
- ○ No
- ○ Significance testing not reported
- ○ Unclear/not described

Job satisfaction

Summarize job satisfaction findings.

____

Copy and paste main job satisfaction findings from text.

____

Was the change in job satisfaction statistically significant?
If there are multiple outcomes, respond "yes" if at least one is siginificant.

- ◯ Yes
- ◯ No
- ◯ Significance testing not reported
- ◯ Unclear/not described

Workload

Summarize workload findings.

____

Copy and paste main workload findings from text.

____

Was the change in workload statistically significant?
If there are multiple outcomes, respond "yes" if at least one is siginificant.

- ◯ Yes
- ◯ No
- ◯ Significance testing not reported
- ◯ Unclear/not described

Other health worker outcomes

Summarize other health worker outcomes findings.

____

Copy and paste main other health worker outcomes findings from text.

____

Was the change in other health worker outcomes statistically significant?
If there are multiple outcomes, respond "yes" if at least one is siginificant.

- ◯ Yes
- ◯ No
- ◯ Significance testing not reported
- ◯ Unclear/not described

Team Outcomes

Team Outcomes How is this outcome assessed? Specific outcome Is there an assessment of change, and what did it show?

Team attitudes

______ ______ ______

Team perceptions

______ ______ ______

Team communication

______ ______ ______

Other teamwork outcome

______ ______ ______

Team attitudes

Summarize team attitudes findings.

____

Copy and paste main team attitudes findings from text.

____

Was the change in team attitudes statistically significant?
If there are multiple outcomes, respond "yes" if at least one is siginificant.

- ◯ Yes
- ◯ No
- ◯ Significance testing not reported
- ◯ Unclear/not described

Team perceptions

Summarize team perceptions findings.

____

Copy and paste main team perceptions findings from text.

____

Was the change in team perceptions statistically significant?
If there are multiple outcomes, respond "yes" if at least one is siginificant.

- ◯ Yes
- ◯ No
- ◯ Significance testing not reported
- ◯ Unclear/not described

Team communication

Summarize team communication findings.

____

Copy and paste main team communication findings from text.

____

Was the change in team communication statistically significant?
If there are multiple outcomes, respond "yes" if at least one is siginificant.

- ◯ Yes
- ◯ No
- ◯ Significance testing not reported
- ◯ Unclear/not described

other teamwork outcome

Summarize other teamwork outcome findings.

____

Copy and paste main other teamwork outcome findings from text.

____

Was the change in other teamwork outcome statistically significant?
If there are multiple outcomes, respond "yes" if at least one is siginificant.

- ◯ Yes
- ◯ No
- ◯ Significance testing not reported
- ◯ Unclear/not described

Service Outcomes

Service Outcomes How is this outcome assessed? Specific outcome Is there an assessment of change, and what did it show?

Safety events

______ ______ ______

Medical errors

______ ______ ______

Hospital-acquired infections

______ ______ ______

Timeliness (reduced wait times)

______ ______ ______

Equity

______ ______ ______

Efficiency

______ ______ ______

Other service outcomes

______ ______ ______

Safety events

Summarize safety events findings.

____

Copy and paste main safety events findings from text.

____

Was the change in safety events statistically significant?
If there are multiple outcomes, respond "yes" if at least one is siginificant.

- ◯ Yes
- ◯ No
- ◯ Significance testing not reported
- ◯ Unclear/not described

Medical errors

Summarize medical errors findings.

____

Copy and paste main medical errors findings from text.

____

Was the change in medical errors statistically significant?
If there are multiple outcomes, respond "yes" if at least one is siginificant.

- ◯ Yes
- ◯ No
- ◯ Significance testing not reported
- ◯ Unclear/not described

Hospital-acquired infections

Summarize hospital-acquired infections findings.

____

Copy and paste main hospital-acquired infections findings from text.

____

Was the change in hospital-acquired infections statistically significant?
If there are multiple outcomes, respond "yes" if at least one is siginificant.

- ◯ Yes
- ◯ No
- ◯ Significance testing not reported
- ◯ Unclear/not described

Timeliness (reduced wait times)

Summarize Timeliness (reduced wait times) findings.

____

Copy and paste main Timeliness (reduced wait times) findings from text.

____

Was the change in timeliness (reduced wait times) statistically significant?
If there are multiple outcomes, respond "yes" if at least one is siginificant.

- ◯ Yes
- ◯ No
- ◯ Significance testing not reported
- ◯ Unclear/not described

Equity

Summarize equity findings.

____

Copy and paste main equity findings from text.

____

Was the change in equity statistically significant?
If there are multiple outcomes, respond "yes" if at least one is siginificant.

- ◯ Yes
- ◯ No
- ◯ Significance testing not reported
- ◯ Unclear/not described

Efficiency

Summarize efficiency findings.

____

Copy and paste main efficiency findings from text.

____

Was the change in efficiency statistically significant?
If there are multiple outcomes, respond "yes" if at least one is siginificant.

- ◯ Yes
- ◯ No
- ◯ Significance testing not reported
- ◯ Unclear/not described

Other service outcomes

Summarize other service outcomes findings.

____

Copy and paste main other service outcomes findings from text.

____

Was the change in other service outcomes statistically significant?
If there are multiple outcomes, respond "yes" if at least one is siginificant.

- ◯ Yes
- ◯ No
- ◯ Significance testing not reported
- ◯ Unclear/not described

Educational Outcomes

Educational Outcomes How is this outcome assessed? Specific outcome Is there an assessment of change, and what did it show?

Trainee learning

______ ______ ______

Other educational outcome

______ ______ ______

Trainee learning

Summarize trainee learning findings.

____

Copy and paste main trainee learning findings from text.

____

Was the change in trainee learning statistically significant?
If there are multiple outcomes, respond "yes" if at least one is siginificant.

- ◯ Yes
- ◯ No
- ◯ Significance testing not reported
- ◯ Unclear/not described

other educational outcomes

Summarize other educational outcomes findings.

____

Copy and paste main other educational outcomes findings from text.

____

Was the change in other educational outcomes statistically significant?
If there are multiple outcomes, respond "yes" if at least one is siginificant.

- ◯ Yes
- ◯ No
- ◯ Significance testing not reported
- ◯ Unclear/not described

Other Outcome types

Other Outcomes How is this outcome assessed? Specific outcome Is there an assessment of change, and what did it show?
Other outcome

______ ______ ______

other outcomes

Summarize other outcomes findings.

____

Copy and paste main other outcomes findings from text.

____

Was the change in other outcomes statistically significant?
If there are multiple outcomes, respond "yes" if at least one is siginificant.

- ◯ Yes
- ◯ No
- ◯ Significance testing not reported
- ◯ Unclear/not described

Bias Assessment

Mixed Methods Appraisal Tool

Additional guidance related to these questions can be found in the MMAT Tool bookmark

The response 'Can't tell' is for papers that do not report sufficent information to answer 'yes' or 'no' for a criterion or that report unclear information related to this criterion. Please add comments if "Can't tell" is selected.

Screening Questions

Are there clear research questions? ◯ Yes ◯ No ◯ Can't tell

Do the collected data allow to address the research question? ◯ Yes ◯ No ◯ Can't tell

Further appraisal may not be feasible or appropriate when the answer is 'No' or 'Can't tell' to one or both screening questions. If you select "no" or "can't tell" please comment to (1)describe what led you to make that selection and (2) note the extent to which you were/were not able to fill out the rest of the tool.

Screening Question Comments

____
(Required if either item in this section is No or Can't Tell; enter "N/A" if both items are Yes and you have no additional comments)

Qualitative

	Yes	No	Can't tell
Is the qualitative approach appropriate to answer the research question?	◯	◯	◯
Are the qualitative data collection methods adequate to address the research questions?	◯	◯	◯
Are the findings adequately derived from the data?	◯	◯	◯
Is the interpretation of results sufficiently substantiated by data?	◯	◯	◯
Is there coherence between qualitative data sources, collection, analysis and interpretation?	◯	◯	◯

Qualitative Section Comments

____
(Required if any item in this section is No or Can't Tell; enter "N/A" if all items are Yes and you have no additional comments)

Quantitative randomized controlled trials

	Yes	No	Can't tell
Is randomization appropriately performed?	◯	◯	◯
Are the groups comparable at baseline?	◯	◯	◯
Are there complete outcome data?	◯	◯	◯
Are outcome accessors blinded to the intervention provided?	◯	◯	◯
Did the participants adhere to the assigned intervention?	◯	◯	◯

Quantitative Randomized Trial Section Comments

____
(Required if any item in this section is No or Can't Tell; enter "N/A" if all items are Yes and you have no additional comments)

Quantitative non-randomized interventional

	Yes	No	Can't tell
Are the participants representative of the target population?	◯	◯	◯
Are the measurements appropriate regarding both the outcome and intervention (or exposure)?	◯	◯	◯
Are there complete outcome data?	◯	◯	◯
Are the confounders accounted for in the design and analysis?	◯	◯	◯
During the study period, is the intervention administered (or exposure occurred) as intended?	◯	◯	◯

Quantitative non-randomized Section Comments

____
(Required if any item in this section is No or Can't Tell; enter "N/A" if all items are Yes and you have no additional comments)

Quantitative descriptive

	Yes	No	Can't tell

Is the sampling strategy relevant to address the research questions?	○	○	○
Is the sample representative of the target population?	○	○	○
Are the measurements appropriate?	○	○	○
Is the risk of nonresponse bias low?	○	○	○
Is the statistical analysis appropriate to answer the research question?	○	○	○

Quantitative Descriptive Section Comments

____
(Required if any item in this section is No or Can't Tell; enter "N/A" if all items are Yes and you have no additional comments)

Mixed methods

	Yes	No	Can't tell
Is there an adequate rationale for using a mixed methods design to address the research question?	○	○	○
Are the different components of the study effectively integrated to answer the research question?	○	○	○
Are the outputs of the integration of qualitative and quantitative components adequately addressed?	○	○	○
Are divergences and inconsistencies between quantitative and qualitative results adequately addressed?	○	○	○
Do the different components of the study adhere to the quality criteria of each tradition of methods involved?	○	○	○

Mixed Methods Section Comments

____
(Required if any item in this section is No or Can't Tell; enter "N/A" if all items are Yes and you have no additional comments)
